# Haemorrhagic Tarlov cyst: A rare complication of anticoagulation therapy

**DOI:** 10.1093/omcr/omab063

**Published:** 2021-08-13

**Authors:** W C Soon, R Sun, M Czyz

**Affiliations:** Department of Neurosurgery, Queen Elizabeth Hospital Birmingham, Birmingham, UK

**Figure 1:**
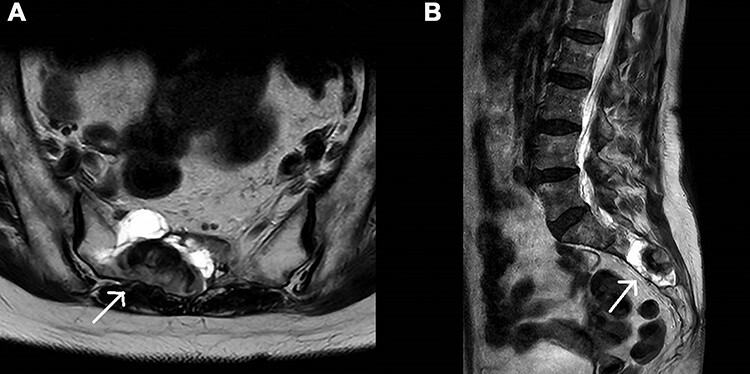
**A and B.** Axial and sagittal T2-weighted MRI of the lumbosacral spine demonstrating the right-sided haemorrhagic Tarlov cyst, as indicated by white arrows.

Tarlov cysts are fluid-filled dilatations of the nerve root sheaths that are present in up to 4.18% of the population [1]. They are mostly asymptomatic and discovered incidentally [2]. We present a rare case of cauda equina syndrome resulting from haemorrhage within a Tarlov cyst. To our knowledge, only one case of spontaneous resolution has been reported in literature prior to this [2].

A 69-year-old woman with no significant past medical history presented with sudden onset lower back pain, perineal numbness and urinary retention. Six days earlier, she was commenced on Rivaroxaban, a direct oral anticoagulant agent for acute unprovoked bilateral pulmonary emboli. Her observations were stable and examination revealed reduced anal tone and decreased sensation in the right S2, 3 and 4 dermatomal distributions.

Magnetic resonance imaging of the lumbosacral spine revealed a 3.4 × 2.0 × 4.7-cm right-sided haemorrhagic multiloculated Tarlov cyst (Figs 1A and B). As per haematology advice, Rivaroxaban was switched to low-molecular weight heparin for a period of four weeks. She remained neurologically stable and was treated non-operatively, due to increased risk for general anaesthesia. Interval imaging showed progressive improvement in the haemorrhagic component of the cyst, she had complete recovery of bladder function and was ultimately commenced on long-term Apixaban in the community.

Patients with symptomatic Tarlov cysts may present with back pain, sensory and/or motor radiculopathy, bladder, bowel and/or sexual dysfunction [3]. CT-guided aspiration and injection of fibrin sealant has been shown to be effective treatment options [4]. There is no clear consensus on the appropriate surgical management of Tarlov cysts and evidence in the literature is limited to small case series [5].

This case highlights a rare complication of anticoagulation, which required prompt recognition and multidisciplinary management. Anticoagulation rationalisation and timely intervention should be initiated in the event of neurological deterioration.

## CONFLICT OF INTEREST STATEMENT

The authors confirm that they have no conflict of interests.

## FUNDING

None.

## ETHICAL APPROVAL

No ethical approval was needed.

## CONSENT

Written consent for publication has been obtained from the patient.

## GUARANTOR

Wai Cheong Soon.
